# Minimal Residual Disease Assessment in the Context of Multiple Myeloma Treatment

**DOI:** 10.1007/s11899-016-0308-3

**Published:** 2016-02-22

**Authors:** Taiga Nishihori, Jinming Song, Kenneth H. Shain

**Affiliations:** Department of Blood and Marrow Transplantation, Moffitt Cancer Center, Tampa, FL USA; Department of Oncologic Sciences, Moffitt Cancer Center/University of South Florida Morsani College of Medicine, Tampa, FL USA; Department of Hematopathology, Moffitt Cancer Center, Tampa, FL USA; Department of Malignant Hematology, Moffitt Cancer Center, Tampa, FL USA; Tumor Biology Department, Moffitt Cancer Center, Tampa, FL USA; Department of Malignant Hematology, H. Lee Moffitt Cancer Center and Research Institute, 12902 Magnolia Drive, Tampa, FL 33612 USA

**Keywords:** Flow cytometry, Allele-specific oligonucleotide, Polymerase chain reaction, Next-generation sequencing, Imaging

## Abstract

With contemporary therapeutic strategies in multiple myeloma, heretofore unseen depth and rate of responses are being achieved. These strategies have paralleled improvements in outcome of multiple myeloma patients. The integration of the next generation of proteasome inhibitors and antibody therapeutics promise continued improvements in therapy with the expectation of consistent depth of response not quantifiable by current clinical methods. As such, there is a growing need to develop adequate tools to evaluate deeper disease response after therapy and to refine the response criteria including the minimal residual disease. Several emerging techniques are being evaluated for these purposes including multi-parameter flow cytometry, allele-specific oligonucleotide polymerase chain reaction, next-generation sequencing, and imaging modalities. In this review, we highlight the recent developments and evaluate advantages and limitations of the current technologies to assess minimal residual disease. We also discuss future applications of these methodologies in potentially guiding multiple myeloma treatment decisions.

## Introduction

Multiple myeloma is a heterogeneous plasma cell neoplasm which remains all but incurable despite recent significant advances in its treatment with various proteasome inhibitors [1], immunomodulatory agents, monoclonal antibodies [2, 3], histone deacetylase inhibitors [4, 5], and widespread use of high-dose melphalan followed by autologous hematopoietic cell transplantation (HCT) and maintenance therapy [6–11]. Multiple myeloma is a unique neoplasm where disease activity and burden can be quantified by a number of different modalities including biochemical analysis (protein or light chain quantification as surrogate markers), bone marrow plasma cell involvement, and functional imaging such as positron emission tomography (PET) [12]. Whole body magnetic resonance imaging (MRI) has also been evaluated for assessing response with negativity after autologous HCT associated with improved prognosis [13]. A number of studies have indicated that deeper responses and achievement of complete response (CR) can serve as a treatment benchmark for longer disease control and survival [14–16].

With increasing number of available therapeutic options in myeloma, probability of achieving CR and stringent CR (sCR) is steadily improving as shown in a recent frontline phase 1/2 study where a combination of carfilzomib, lenalidomide, and dexamethasone resulted in unprecedented sCR rate of 42 % with early clinical trials suggesting even greater depths of response in the near future [17]. However, these excellent responses are unfortunately not always sustained and many patients (if not all) experience disease progression or relapse primarily due to residual myeloma reservoir through environment-mediated drug resistance and/or the persistence of myeloma stem cells [18, 19]. This underscores the importance of defining further and deeper response beyond sCR.

We have reached another important era in multiple myeloma with expanding treatment options. To this end, our ability to quantify low-level disease burden and our response criteria must keep pace. Several sensitive technologies to assess minimal residual disease (MRD) in myeloma have been developed and are clinically available although no consensus exists on how and when to use these newer methods for the detection and monitoring of MRD. In this review, we focus on technologies such as multi-parameter flow cytometry (FCM), polymerase chain reaction (PCR), and next-generation sequencing (NGS) to assess MRD burden in the context of multiple myeloma treatment.

## Refinement of CR Criteria by International Myeloma Working Group

With the growing knowledge on MRD assessment, the updated International Myeloma Working Group (IMWG) response criteria in 2011 incorporated some new designations to traditional CR definitions [16]. “Immunophenotypic CR” is defined as absence of phenotypically aberrant clonal plasma cells in bone marrow with a minimum total of one million bone marrow cells analyzed by multi-parameter FCM (with ≥4 colors). Additionally, “molecular CR” is now defined as confirmation of CR plus negative allele-specific oligonucleotide PCR (sensitivity 10^−5^) [16]. This represents a step forward in adapting new and emerging technologies to official response criteria. Whether these endpoints will be proven to be the clinical relevant endpoints [20] remains to be seen, and the IMWG response criteria are expected to evolve even more in the near future. In the next sections, we will review the technologies being assessed for MRD quantification at this time as well as potential future options.

## Multi-Parameter Flow Cytometry

### Principles of FCM in Myeloma

Multi-parameter FCM is probably the most commonly available technique that allows quantifications of MRD assessment. The technique employs different cocktails of antibodies targeting unique plasma cell surface protein expression enabling differentiation of normal versus abnormal (or aberrant) plasma cells in the bone marrow aspirate samples. Almost a decade ago, the European Myeloma Network (EMN) convened FCM workshops for myeloma and reported a list of most useful antigens for the detection of aberrant plasma cells [21]. In addition to CD38, CD138, and CD45, markers such as CD19 (negative) and CD56 (positive) were considered essential for detection of abnormal plasma cells. The panel recommended additional antibodies to be included such as CD117, CD20, CD28, and CD27 for prognostication. Although data were limited, CD81 and CD200 were also suggested to be also included [21].

**Fig. 1:**
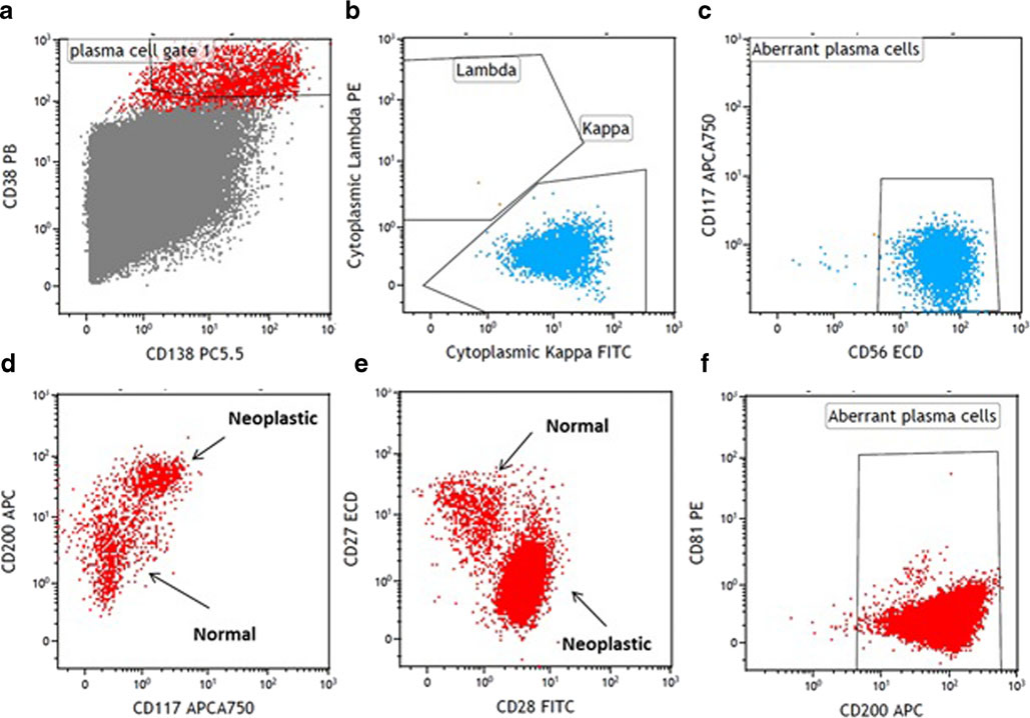
Characterization of neoplastic plasma cells by multi-parameter flow cytometry. The plasma cells are identified by gating on CD38+ (strong) and CD138+ cells (**a**). The neoplastic plasma cells are detected by light chain restriction (**b**), aberrant expression of CD56 (**c**), or CD117 (**d**), increased expression CD200 (**d**, **f**) or CD28 (**e**), and decreased expression of CD27 (**e**) or CD81 (**f**). Note: the demonstration shown is compiled from different patients

Figure 1 shows some representative aberrant immunophenotype of neoplastic plasma cells. The development and use of therapeutic anti-CD38 antibodies, such as daratumumab [22], which could reduce the expression of CD38 on the plasma cells, has also made it necessary to search for alternative markers for the identification of normal or neoplastic plasma cells. CD269, CD319, CD229, and CD54 have been found to be valuable for this purpose. CD269 and CD319 were found to identify plasma cells under substantially more diverse conditions than CD138, including within delayed samples [23].

Multi-parameter FCM is generally applicable to majority of myeloma patients with a sensitivity ranging from 10^−4^ to 10^−5^ (1 in 10,000 to 100,000 cells) [21, 24–26]. However, expertise is required to construct adequate antibody panel for multi-parameter FCM for MRD assessment in myeloma. Each center and FCM laboratory may choose desired antibody cocktails and fluorochromes of their choices and the panels would need to be validated prior to clinical use. Consistent application of FCM technique and interpretation of FCM analysis by experienced pathologists are also essential for reliable reporting of MRD in myeloma. Many colors as much as 10 (i.e., 10-color flow panel) are available in recent flow cytometer expanding the ability to simultaneously target multiple antibodies for FCM analysis.

Using the principles outlined by the EMN [21], Medical Research Council (MRC) Myeloma IX Study performed the multi-parameter FCM in a single laboratory (in Leeds, UK) where a six-color panel of CD138, CD45, CD38, CD19, CD56, and CD27 was used [27••]. This study demonstrated the predictive ability of multi-parameter FCM performed at 100 days after autologous HCT [27••]. In an effort to standardize the flow panel, the EuroFlow (a division of European Scientific foundation for Laboratory HematoOncology, www.euroflow.org) reported a validated eight-color flow panel for myeloma FCM [28]. They proposed two tubes to be used for the eight-color panel FCM: tube 1 (CD45/CD138/CD38/CD56/beta-2 microglobulin/CD19/cytoplasmic Ig kappa/cytoplasmic Ig lambda) and tube 2 (CD45/CD138/CD38/CD28/CD27/CD19/CD117) [28]. These panels are designed to use four backbone markers (CD38/CD138/CD45/CD19) and eight additional markers for further identification, enumeration, and characterization of neoplastic plasma cells.

### Monitoring of MRD with FCM After Treatment

At least two large studies evaluated the predictability of multi-parameter FCM on multiple myeloma treatment (Table 1). Paiva et al. assessed MRD status at day +100 by multi-parameter FCM in 295 newly diagnosed multiple myeloma patients treated with induction followed by autologous HCT in the GEM2000 protocol [29]. Compared to MRD-positive patients at day +100, those with MRD-negative status had longer progression-free survival (PFS; median 71 vs. 37 months, *P* < 0.001) and overall survival (OS; median not reached vs. 89 months, *P* = 0.002: Table 1) [29]. In multivariate analyses, MRD status by multi-parameter FCM at day +100 after autologous HCT was the most important independent prognostic factor for both PFS (hazard ratio (HR) = 3.64, *P* = 0.002) and OS (HR = 2.02, *P* = 0.02).

**Table 1:** Selected large-scale prospective studies incorporating multi-parameter FCM for multiple myeloma

Study/author	Myeloma patient population/treatment	MRD assessment/sensitivity	Treatment outcomes (based on MRD status at day +100 after autologous HCT)		
			Survivals	MR−	MRD+
GEM (Grupo Español Multidisciplinar de Melanoma) 2000 protocol/Paiva et al. [29]	VBMCP/VBAD induction followed by autologous HCT (*n* = 295)	4-Color panels: •CD38/CD56/CD19/CD45 •CD138/CD28/CD33/CD38 •CD20/CD117/CD138/CD38 Sensitivity: 0.01 % or 10^−4^ (3 × 10^5^ events for second acquisition)	•Median PFS	71 months	37 months (*P* < 0.001)
			•Median OS	Not reached	89 months (*P* = 0.002)
			•5 years PFS (in CR patients (*n* = 147))	62 %	30 % (*P* < 0.001)
			•5 years OS (in CR patients (*n* = 147))	87 %	59 % (*P* = 0.009)
MRC Myeloma IX trial/Rawstron et al.	CVAD or CTD followed by autologous HCT ± thalidomide maintenance (intensive pathway, *n* = 397)	6-Color panel: •CD138/CD45/CD38/CD19/CD56/CD27 Sensitivity: 0.01 % or 10^−4^ (5 × 10^5^ events for post HCT samples)	•Median PFS	28.6 months	15.5 months (*P* < 0.001)
			•Median OS	80.6 months	59 months (*P* = 0.0183)
			•Median PFS (in CR patients)	34.4 months	14.1 months (*P* = 0.0068)
			•Median OS (in CR patients)	Not reached	61.9 months (*P* = 0.0928)

*CR* complete response, *CTD* cyclophosphamide, thalidomide, and dexamethasone, *CVAD* cyclophosphamide, vincristine, doxorubicin, dexamethasone, *FCM* flow cytometry, *GEM* Grupo Español Multidisciplinar de Melanoma, *HCT* hematopoietic cell transplantation, *MRD* minimal residual disease, *n* number, *OS* overall survival, *PFS* progression-free survival, *VBMCP/VBAD* carmustine, melphalan, cyclophosphamide, prednisone/vincristine, carmustine, doxorubicin, dexamethasone

Rawstron et al. evaluated the role of multi-parameter FCM in assessing MRD after induction therapy (*n* = 378) and at day +100 after autologous HCT (*n* = 397) in MRC Myeloma IX Study [27••]. MRD status at day +100 after autologous HCT was highly predictive of the survival: median PFS 28.6 vs. 15.5 months (*P* < 0.001) and median OS 80.6 vs. 59 months (*P* = 0.0183) for MRD-negative and MRD-positive patients, respectively (Table 1) [27••]. In both studies for patients achieving CR, MRD-negative status was associated with better PFS but not with OS in MRC Myeloma IX Study [27••, 29]. In the MRC Myeloma XI trial, the best outcome was noted in patients with favorable cytogenetics and MRD-negative status with the worse outcome seen in patient with adverse cytogenetics and MRD-positive status for both PFS and OS (*P* < 0.001) [27••]. Although some studies appear to show prognostic values of MRD status for survivals in high-risk myeloma population, it is important to note that further improvement in treatment options and/or strategies is clearly needed for high-risk disease due to overall inferior prognosis.

### Limitations of Multi-Parameter FCM

Although multi-parameter FCM is likely the most widely available technique with excellent sensitivity, a number of potential issues may limit its application. A major limitation of FCM is the requirement for quality bone marrow sampling. Other institutional level limitations may include the lack of standardization of FCM protocols and variability in sensitivity, panels, and performance among the pathology and/or FCM laboratories. Roschewski et al. surveyed 27 academic institutions in the USA and reported wide variations in MRD assessment practices in 2014 [30]. Among the 11 institutions that do perform FCM, there was a considerable heterogeneity in its methodology including antibody panels and sensitivity of multi-parameter FCM [30]. There is an ongoing need to have an accepted, uniform, and standardized FCM methodology for MRD analysis in myeloma which would help improve overall quality of care provided to myeloma patients and facilitate the advancement of MRD assessment technology.

In addition, first-generation FCM techniques lacked the sensitivity of allele-specific oligonucleotide (ASO) PCR and NGS. These initial FCM assays allowed reproducible detection limits of 10^−4^ and more recently to 10^−5^. However, recent reports from the EuroFlow Consortium and International Myeloma Foundation indicate that even more sensitive assessment is feasible with novel design and the utilization of larger number of cells (e.g., next-generation FCM) [31]. Further, although in a small subset analysis, the authors demonstrated that next-generation FCM outperformed NGS [31]. These suggest that the sensitivity of multi-parameter FCM may be able to approach that of the molecular techniques.

## Polymerase Chain Reaction (Using Allele-Specific Oligonucleotide)

### Principles of ASO-PCR in Myeloma

ASO-PCR was developed as a means to detect the unique (clonal) rearrangements in the V(D)J-junctional regions of the germline Ig genes in lymphoid cells. As such, these regions provide veritable fingerprints for individual patients [32•]. For nearly two decades, the concept of ASO-PCR has evolved from nested primers to real-time quantitative PCR to quantitate the disease burden (MRD status) relative to a diagnostic sampling in a number of B cell malignancies including myeloma [33, 34]. As a mature B cell malignancy, multiple myeloma has also undergone somatic hypermutation which further alter the V(D)J regions. These highly mutated regions have mitigated the widespread utilization of ASO-PCR being applicable to a limited number of myeloma patients [32•]. However, the use of parallel quantification of IgH (heavy chain) and IgL (light chain) as well as unmutated Ig rearrangements has been utilized to decrease the false discovery rates [35, 36].

PCR for ASO has been thought to be the most sensitive methodology for the detection of abnormal plasma cells with potential sensitivity limit of 10^−5^ to 10^−6^. In a small study (*n* = 14), patients who achieved CR after allogeneic HCT were assessed for ASO-PCR [37]. Seven patients (50 %) who achieved molecular CR (negative ASO-PCR) did not relapse after a median follow-up of 60 months [37]. In contrast, only one out of other seven patients who never achieved molecular CR relapsed suggesting a very high sensitivity of the assay [37]. Martinez-Sanchez et al. utilized fluorescent-PCR to assess IgH incomplete rearrangements (DHJ) and light chain genes to overcome problems of somatic mutations in JH segments for MRD assessment in GEM2000 protocol [38]. Fluorescent-PCR was feasible in 91 % of patients, and the results were overall similar to FCM. Multivariate analysis showed fluorescent-PCR to be the only variable for prognostic significance for PFS [38].

### Monitoring of MRD with ASO-PCR After Treatment

MRD-negative status defined by ASO-PCR has been shown to correlate with improved patient outcomes. Although a relatively small study, Ladetto et al. evaluated 39 patients receiving bortezomib, thalidomide, and dexamethasone consolidation after autologous HCT with real-time quantitative PCR [39]. Molecular remission defined by real-time quantitative PCR improved from 3 % after autologous HCT to 18 % after bortezomib, thalidomide, and dexamethasone consolidation [39]. The sensitivity of this method was reported at 5 × 10^−6^ and, importantly, no patients who achieved molecular remission had progressed after a median follow-up of 42 months. This study documents the possibility of achieving molecular response with consolidation therapy after autologous HCT without performing allogeneic HCT [39].

Puig et al. compared real-time quantitative ASO-PCR and multi-parameter FCM in three consecutive Spanish myeloma trials enrolling 170 patients [40••]. The applicability of PCR technique was limited to 42 % of cases due to lack of clonality detection, unsuccessful sequencing, and suboptimal ASO performance [40••]. When MRD status was assessed in 103 patients, there was a significant correlation in MRD quantification for both techniques and patients with <10^−4^ residual myeloma cells had longer PFS compared to the other (median PFS not reached vs. 31 months, *P* = 0.002) [40••]. The study underscores the strength of real-time quantitative ASO-PCR for higher sensitivity; however, it also uncovered its limited applicability and multi-parameter FCM deems to be a more practical method for MRD assessment.

### Limitations of ASO-PCR

ASO-PCR represents a novel technology with high sensitivity for assessing the presence of MRD in myeloma. However, ASO-PCR does carry potential limitations. The widespread applicability ASO-PCR has been attenuated by cost, extended turnaround time, and a need for a design of a patient-specific ASO primer (requiring a diagnostic sample). The applicability of ASO-PCR in myeloma is made more difficult as a consequence of somatic hypermutation which further alter the V(D)J regions. These highly mutated regions alter sequence and thus increase the difficulty of designing primers for quantification making ASO-PCR applicable to only 60–70 % of myeloma patients [32•, 40••]. However, the use of alternate PCR techniques continues to improve the applicability [35, 36]. Any subsequent gene mutations (i.e., tumor evolution) would invalidate the utility of Ig derived from an original sample. Taken together, these data demonstrate that although both the sensitivity and specificity may be quite high when patient-specific ASO-PCR is performed, difficulty in generalizability of this technique hampers its dissemination in clinical practice [41•].

## Next-Generation Sequencing

### Principles of NGS in Myeloma

With rapid technological advances, NGS has been applied to many lymphoid malignancies as a deep sequencing method for monitoring of disease activity and assessment of MRD [41•–45]. NGS employs multiplex sequencing of immunoglobulin heavy chain (IgH) for B lymphocytes and plasma cells. Typically, diagnostic sample from the initial biopsy (either bone marrow or plasmacytoma) would be required to identify unique IgH rearrangements present in the original clone. It is possible that several clones may be discovered from the diagnostic sample. Using the information obtained from the diagnostic sample, subsequent samples both in bone marrow and peripheral blood could be assessed to identify amplified IgHs. The sensitivity of NGS using IgH sequences may approach 10^−6^ or better. Once clonal patterns of individual malignancy are established, these can be monitored over the course of therapy. The use of NGS in multiple myeloma provides an exciting avenue to utilize a highly sensitive and specific platform that may overcome some of the limitations of ASO-PCR techniques.

### Monitoring of MRD with NGS After Treatment

Martinez-Lopez et al. evaluated the prognostic value of NGS method for MRD assessment using a sequencing-based platform in 133 multiple myeloma bone marrow samples from patients treated under various GEM protocols and achieved at least very good partial response (VGPR) after frontline therapy [46••]. High-throughput sequencing (HTS) was performed by amplifying genomic DNA using locus-specific primer sets for the IgH locus complete (IgH-VDJ_H_), IgH incomplete (IgH-DJ_H_), and immunoglobulin kappa (IGK) locus [46••]. The MRD was defined as <10^−5^ and the assessment by NGS was applicable in 91 % of patients. MRD-negative status by HTS was associated with significantly longer time to progression (TTP) (median 80 vs. 31 months, *P* < 0.0001) and OS (median not reached vs. 81 months, *P* = −.02) [46••]. Deeper response appears to show better disease control where median TTP was 27 vs. 48 vs. 80 months for MRD levels ≥10^−3^ vs. 10^−3^ to 10^−5^ vs. <10^−5^ (*P* values from 0.003 to 0.0001), respectively [46••]. When compared with other MRD methodologies, concordance rates between NGS and multi-parameter FCM, and NGS and ASO-PCR were 83 and 85 %, respectively [46••]. In patients who achieved CR, MRD-negative status by NGS resulted in significantly longer TTP (median 131 vs. 35 months, *P* = 0.0009). This study illustrates the powerful potential of NGS method in assessing MRD and its correlation with clinical outcomes.

### Limitations of NGS

Although NGS provides what appears to be increased sensitivity and applicability to myeloma patients, it too has limitations. NGS can be limited in its ability to capture a clonotype in all samples in spite of adequate samples [41•]. NGS remains the least MRD testing modality studied and continued clinical correlations will be required for standardization and utilization. Lastly, NGS may be susceptible to the heterogeneity or the evolving heterogeneity of myeloma with therapy. Over 37 % of myeloma patients will contain multiple evolved clonotypes [45]. Further, with therapy, new immunoglobulin clonotypes may emerge, suggesting ongoing mutational processes that could limit the sequential testing of NGS without clear appreciation of the clonal dynamics.

## Imaging in the Assessment of MRD

One of the foreseen limitations of the MRD assessment techniques outlined thus far is the dependence on bone marrow sampling. It has long been perceived that multiple myeloma is a patchy rather than diffusely infiltrative marrow process. As such, the accuracy of multi-parameter FCM, ASO-PCR, and NGS testing for MRD assessment may be attenuated by this nature of myeloma marrow infiltration pattern. To address this issue in part, highly sensitive imaging techniques with PET/CT and MRI may represent plausible solutions.

### MRI

Historically, the assessment of multiple myeloma bone disease was carried out via skeletal surveys which are at best only able to capture macroscopic disease. Fortunately, contemporary imaging techniques are able to provide a much greater sensitivity. MRI represents a highly sensitive and non-invasive modality to assess intramedullary and extramedullary diseases. Multiple myeloma lesions are typically identified as low-signal intensity on T1-weighted imaging and high-signal intensity on T2-weighted imaging. Whole body MRI have been demonstrated to be prognostic demonstrating that an increased number of focal lesions correlated with an increased rate of progression from smoldering myeloma to symptomatic disease [13, 47].

Importantly, the sensitive nature of MRI also positions it as an additional modality to assess MRD status. A number of retrospective analyses have demonstrated that resolution of MRI-positive lesions after autologous HCT correlates with improved outcomes [48, 49]. These data demonstrate the high potential of MRI as a medium for assessing MRD status. However, MRI does have limitations. First, the whole body MRI is not widely available. Second, MRIs are time consuming and individuals with pacemakers or other non-compatible hardware are not able to undergo imaging via this technique. Third, although quite sensitive, MRI does not differentiate between active and inactive lesions after therapy; as such, it is important to ensure that 3 months have elapsed to allow for resolution of MRI-positive lesions after the therapy [13].

### PET/CT

PET/CT imaging represents an ideal combination of structural and activity-based assessment of skeletal-related events. Importantly, PET/CT also affords accurate characterization of extramedullary disease, thus taking into account intramedullary and extramedullary processes [50]. Paiva et al. described that in a comparison of transplant-eligible patients, PET/CT was demonstrated to provide equivalent sensitivity to whole body MRI; however, a greater specificity was observed [32•]. Another study suggested that PET/CT was superior to MRI on whole body analysis, whereas in the spine and pelvic, MRI was comparable to PET/CT [51]. Collectively, these results highlight the potential of PET/CT as an important structural and activity-based assessment of MRD. Therefore, one could consider PET/CT as a routine assessment of disease response and activity.

## MRD Testing as a New Standard of Care

The growing data indicate that depth of response correlates with improved outcomes in multiple myeloma. In a long-term follow-up study, Martinez-Lopez et al. demonstrated that myeloma patients attaining a CR after autologous HCT (before the novel anti-myeloma agent era) have improved outcomes relative to those not achieving those levels of response [52]. Similarly, it is presently assumed that achieving CR may be a prerequisite for long-term survival in the era of novel agents [53]. This experience has also been replicated in the Total Therapy program at the University of Arkansas where increased depth of response has been linked to long-term survival and possibly operational cure of myeloma [54, 55]. These data suggest that CR at minimum should be the goal of therapy for all myeloma patients. Recent studies evaluating the contemporary combination therapy using carfilzomib/lenalidomide/dexamethasone induction, followed by high-dose therapy and autologous HCT, and subsequently carfilzomib/lenalidomide/dexamethasone consolidation have the potential to further improve the treatment outcomes in the frontline setting [17, 56]. Although the long-term outcomes of the newer regimens remain to be seen, these data illustrate the enormous potential for providing consistently much deeper responses in newly diagnosed multiple myeloma patients; thus, our current serological (and nephelometric) methods of measuring myeloma responses will soon be outdated and defining new response criteria deeper than the conventional CR will be urgently needed.

## Future Directions

Multi-parameter FCM, ASO-PCR, and NGS may be all equally sensitive in identifying MRD; however, they are limited by the mere fact that each is dependent on sampling of bone marrow plasma cells. Therefore, they cannot overcome two major limitations. First, that myeloma is notoriously a patchy disease and bone marrow biopsies are not always representative of systemic disease burden. Second, bone marrow biopsies are an invasive procedure and frequent sampling remains less than ideal in most circumstances. To this end, alternate peripheral blood-based testing would provide important advantages over the discussed MRD testing modalities. Assessment of peripheral blood for the monitoring of MRD may overcome some of the logistical and sampling issues associated with bone marrow testing. Solid tumor studies have demonstrated the presence of circulating tumor cells (CTC), and circulating cell-free DNA may also facilitate the characterization of the genotype of a particular tumor of interest, but not all. The continually improving sensitivity of NGS also highlights the potential of this kind of analysis. The feasibility of accessing CTCs in multiple myeloma has been recently investigated by others. Mishima et al. demonstrated that CTCs could be identified in 80 % of patients with newly diagnosed multiple myeloma using multi-parameter FCM. Further, CTCs were identified in 55 % of patients with relapsed myeloma [57]. Although they were able to characterize the genotype in a number of samples, this level of detection does not translate to MRD testing. Direct assessment of circulating cell-free DNA in myeloma patients has also been examined in the context of MRD testing. In multiple myeloma patients treated with carfilzomib/lenalidomide/dexamethasone, evaluation of circulating DNA has been performed. Circulating DNA was quantifiable in all samples; however, levels became undetectable after only four cycles of therapy [58]. These and others suggest that emerging new assessment tool requires further technological advances to be considered for reliable MRD testing method.

## Conclusion

The addition of the next-generation proteasome inhibitors, histone deacetylase inhibitors, and myeloma-selective antibodies to our armamentarium leads to great expectations. Upfront combination therapies with novel combination regimens without autologous HCT demonstrated near CR/CR rates approximating 31 % after eight cycles of carfilzomib/lenalidomide/dexamethasone [17]. Integrating autologous HCT into carfilzomib/lenalidomide/dexamethasone treatment resulted in near CR/CR rates of 91 % [56]. Although limited in a number of patients and follow-up, Zimmerman et al. demonstrated that 15/17 evaluable patients (88 %) achieved MRD-negative status [56]. Collectively, these results highlight the improving depth of response attainable with future management strategies. As such, our ability to quantitate response needs to evolve as well. Multi-parameter FCM (or next-generation FCM), ASO-PCR, and NGS represent three highly sensitive metrics with which to assess MRD from bone marrow samples. In addition, imaging with PET/CT and MRI represents two critical techniques to assess systemic MRD via contemporary imaging. Continued evaluation will define which of these techniques will be adopted for assessment of MRD (for now) alone or in combination based on generalizability, accuracy, sensitivity, as well as economic considerations [59, 60]. However, achieving MRD-negative status may not offer solutions to all of myeloma clinical challenges and there are limitations. There remain subgroups of myeloma patients in which MRD-negative status may not equate to long-term survival [31, 45, 61].
